# Pediatric traumatic brain injury and later psychotic syndromes in Finland

**DOI:** 10.1007/s00431-025-06224-3

**Published:** 2025-05-31

**Authors:** Juho Laaksonen, Ville Ponkilainen, Julius Möttönen, Ville M. Mattila, Ilari Kuitunen

**Affiliations:** 1 Department of Clinical Medicine, University of Tampere, Tampere, Finland; 2 Department of Orthopedics and Traumatology, Tampere University Hospital, Tampere, Finland; 3 Coxa Hospital for Joint Replacement, Tampere, Finland; 4 Institute of Clinical Medicine and Department of Pediatrics, University of Eastern Finland, Kuopio, Finland; 5 Department of Pediatrics, Kuopio University Hospital, Kuopio, Finland

**Keywords:** Traumatic brain injury, Psychotic syndrome, Pediatric, Trauma

## Abstract

The association between pediatric traumatic brain injury (pTBI) and post-traumatic psychotic syndromes remains understudied subject. The objective of this study was to evaluate the association between pTBI and subsequent psychotic syndrome incidence in Finland. A nationwide retrospective cohort study in Finland from 1998 to 2018 included 71,969 pediatric (< 18 years) TBI patients and 64,856 references with distal extremity fractures. Our primary outcome was psychotic syndrome occurrence post-pTBI, analyzed via Kaplan–Meier (KM) survival and multivariable Cox regression with 95% confidence intervals (CI).. KM analyses demonstrated consistently elevated rates in pediatric TBI (pTBI), particularly notable 10 years post-trauma. Beyond the initial year, cumulative incidence was 0.04% in pTBI and 0.02% in the reference group. Cox regression revealed increased long-term psychotic syndrome risk in pTBI compared to the reference. Up to 10 years of follow-up, the hazard ratio (HR) was 1.34 (95% CI: 1.16–1.55). Notably, among females, pTBI exhibited higher cumulative rates than the reference group. Post the first year, cumulative incidence was 0.06% (pTBI) versus 0.03% (reference) for females. Following 3 years, the HR for pTBI females was 2.00 (95% CI: 1.24–3.23), decreasing to 1.08 (95% CI: 0.90–1.30) from 3 to 20 years.

*Conclusion*: These findings suggest that the risk of psychotic syndromes remains slightly elevated up to 10 years following pTBI.

**Table:** 

**What Is Known:** *• The association between pTBI and post-traumatic psychotic syndromes is relatively understudied. Existing literature mainly focuses on schizophrenia.* **What Is New:** *• This nationwide study found an association between pTBI and an increased risk of post-traumatic psychotic syndromes, particularly within the first 10 years after injury. Notably, female patients faced a higher risk.*

**Supplementary Information:**

The online version contains supplementary material available at 10.1007/s00431-025-06224-3.

## Introduction

The current hypothesis proposes that traumatic brain injury (TBI) sets off an extended secondary injury sequence, driven by factors like neuroinflammation, oxidative stress, and endoplasmic reticulum stress. This sequence ultimately may lead to neurodegeneration, potentially contributing to severe psychiatric problems [1, 2]. The association between pTBI and psychotic syndrome is a relatively unexplored area [3, 4]. Our study aims to fill this knowledge gap, as pTBI is linked to an increased risk of post-traumatic psychotic syndromes overall (HR = 1.8), as well as schizophrenia (incidence rate ratio [IRR] = 1.3) and bipolar disorder (IRR = 1.3) specifically [3, 4]. Additionally, the incidence of pTBI is rising [5, 6].

## Materials and methods

This nationwide retrospective cohort study utilized data from the Finnish Care Register for Health Care and the Finnish Social Insurance Institution, covering January 1998 to December 2018. The primary cohort included individuals under 18 diagnosed for pTBI (ICD-10 S06*). Reference groups comprised < 18 individuals with ankle (ICD-10 S82*) or wrist fractures (ICD-10 S52*). All patients were hospitalized and treated in secondary healthcare units. Hospitalization means that the patient either had an emergency department visit in secondary/tertiarty healthcare unit, or alternatively was admitted to hospital as an inpatient. TBI cases were categorized by treatment to conservatively treated and operatively treated (Appendix 1 and 2).

Psychotic syndrome diagnoses—referring to psychiatric or neurological conditions involving a clear disturbance in the perception of reality—were identified using reimbursement code 112 from the Finnish Social Insurance Institution. This includes conditions such as schizophrenia, bipolar disorder with psychotic features, and psychotic depression, with the date of onset recorded. The reimbursement code covers medications for psychotic syndromes and requires a comprehensive psychiatric assessment provided by a specialized healthcare unit. It is based on ICD-10 diagnostic codes. All individuals diagnosed with a psychotic syndrome in Finland receive this code regardless of treatment adherence, which allowed us to capture all diagnosed cases in the country. There were no exclusion criteria; however, only individuals with a psychotic syndrome diagnosed after the injury were included in the analysis.

### Statistical analysis

Descriptive statistics included frequencies (%) and median with interquartile range (IQR) for the distribution. Only the initial hospitalization was considered for cases with repeated injuries.

The follow-up began from the date of pTBI and it ended to one of four events: psychosis diagnosis, death, emigration or the common closing date of the follow-up (31 st December 2018).

Cumulative incidence of psychotic syndrome diagnoses was evaluated through Kaplan–Meier (KM) analysis and a multivariable Cox regression model in both the pTBI and reference groups with 95% CIs. Covariate selection for the regression analysis was guided by a directed acyclic graph (DAG) [7], which included age at hospitalization and sex (Appendix 4). Subgroup analyses examined sex differences in cumulative incidence of psychotic syndromes within reference and pTBI groups, following the methodology of the main analysis. Porpotional hazards (PH) assumptions were checked continuously using scaled Schoenfeld residuals, with cut-off points set at 10 and 18 years for the main analysis, and at 3 years for the subgroup analysis.

To handle PH violations, a time-dependent coefficients method with a step function was applied [8]. Time axis was divided into 0–9 years, 10–17 years, and 18–20 years intervals in main analysis and into 0–2 years and 3–20 years intervals in subgroup analysis.

Analyses were conducted by R Windows version 4.0.5 (R Foundation for Statistical Computing, Vienna, Austria) with the *survival* package.

### Ethics

The retrospective study design did not require ethical approval according to Finnish guidelines. Rigorous pseudonymization, in compliance with the Personal Data Act, was carried out under the supervision of Statistics Finland. Access to the Care Register was granted by Findata, while Statistics Finland provided access to the Population Information System and the Register of Causes of Death.

## Results

The primary cohort included 71,969 patients with pTBI and 64,856 references. Of these, 4,012 had both conditions and were included in the pTBI group. (Appendix 3).

During follow-up, 1,270 patients were diagnosed with psychotic syndrome post-injury. Median time from trauma to psychotic syndrome was 7.94 years in pTBI group and 9.39 years in references. A total of 734 patients passed away. Median age at hospitalization was 7 years among pTBI, 11 years in reference group. In pTBI group, 58% were males, vs. 62% in references. Among pTBI patients, 337 had operations, with 4 (1.19%) post-injury psychotic syndromes (Table 1).

**Table 1:** Characteristics of patients in relation to psychotic syndrome following pTBI in Finland during the period from 1998 to 2018

	pTBI group	Reference group
Number of patients	71,969	64,856
Median age at the time of trauma in years (IQR)	7 (3–13)	11 (8–14)
Sex, n (%)		
Male	41,496 (58%)	40,111 (62%)
Female	30,473 (42%)	24,745 (38%)
Psychotic syndrome, n (%)	611 (0.85%)	659 (1.02%)
Male	316 (52%)	422 (64%)
Female	295 (48%)	237 (36%)
Median time to psychotic syndrome in years (IQR)	7.94 (4.57–12.00)	9.39 (6.22–12.50)

*TBI*= Traumatic brain injury

*CI*=Confidence interval

*n*= number

**Fig. 1:**
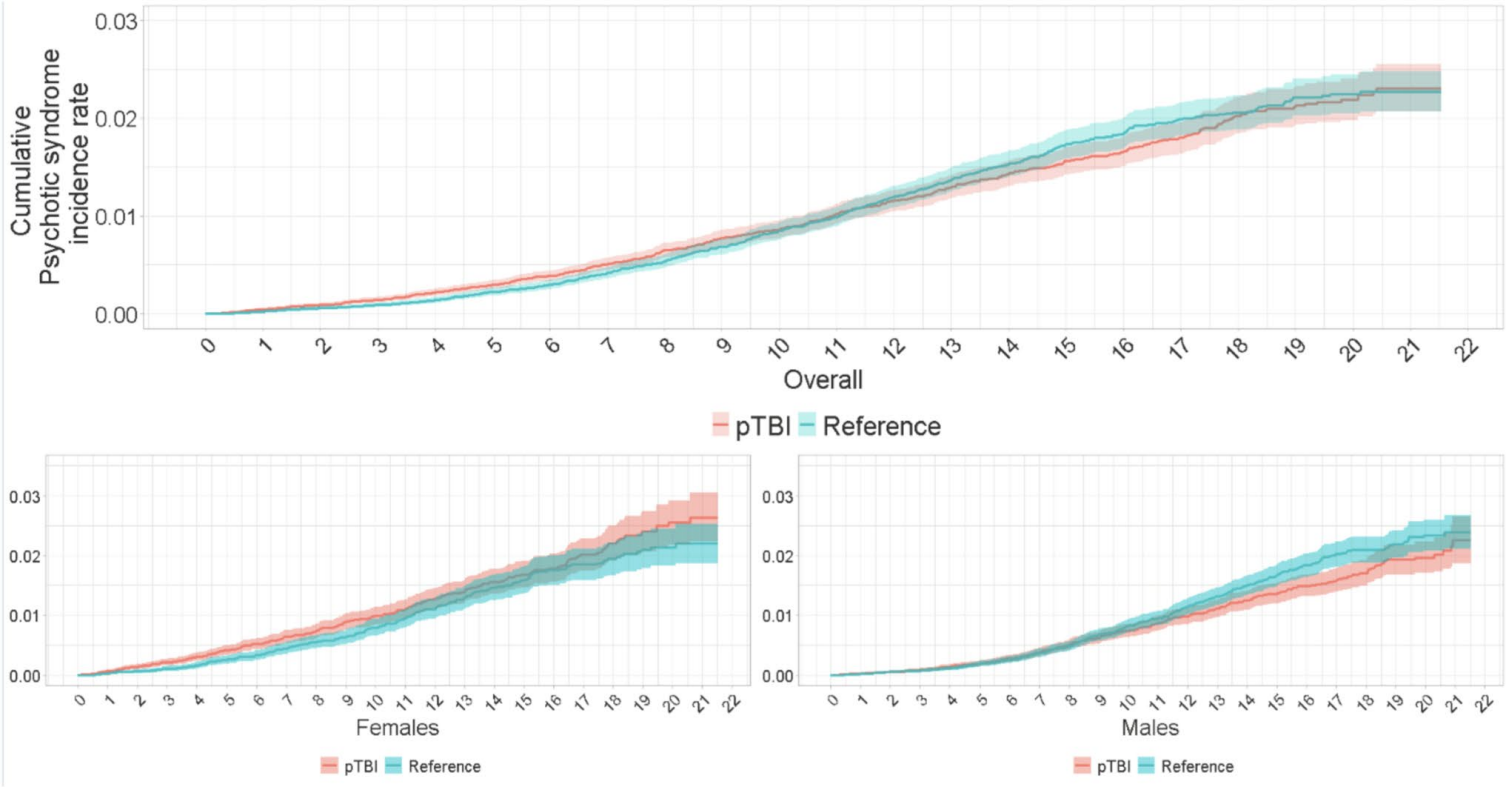
Kaplan–Meier curve depicting the cumulative incidence of psychotic syndrome in the study population, including 95% confidence intervals, among individuals with pediatric traumatic brain injury (pTBI) in Finland from 1998 to 2018

Kaplan–Meier analyses showed consistently elevated cumulative incidence rates for psychotic syndrome in the pTBI group during the initial 10 years of follow-up. Rates were 0.04% (pTBI) vs. 0.02% (reference) after one year, increasing to 0.14% (pTBI) vs. 0.09% (reference) after three years. By 20 years, rates rose to 2.19% (pTBI) vs. 2.25% (reference) (Fig. 1, Table 2).

**Table 2:** Psychotic syndrome at 1–20 years after pediatric trauma in TBI and distal extremity fracture groups in a finnish nationwide sample

	Overall		Females		Males	
	pTBI	Reference	pTBI	Reference	pTBI	Reference
N.of	**71,969**	**64,856**	**30,473**	**24,745**	**41,496**	**40,111**
at 1 year						
n. risk	**66,566**	**61,399**	**28,225**	**23,393**	**38,578**	**38,074**
Cumulative incidence	0.04%	0.02%	0.06%	0.03%	0.03%	0.02%
(CI)	(0.02–0.06)	(0.01–0.03)	(0.03–0.09)	(0.01–0.05)	(0.01–0.04)	(0.01–0.03)
at 3 years						
n. risk	**46,788**	**48,029**	**23,617**	**20,618**	**32,598**	**34,136**
Cumulative incidence	0.30%	0.22%	0.21%	0.11%	0.09%	0.07%
(CI)	(0.25–0.34)	(0.18–0.26)	(0.16–0.27)	(0.06–0.15)	(0.06–0.12)	(0.05–0.10)
at 10 years						
n. risk	**29,332**	**32,987**	**12,285**	**12,304**	**17,790**	**21,377**
Cumulative incidence	0.86%	0.85%	0.99%	0.80%	0.74%	0.83%
(CI)	(0.77–0.95)	(0.76–0.93)	(0.84–1.13)	(0.66–0.93)	(0.63–0.85)	(0.72–0.94)
at 20 years						
n. risk	**3,855**	**4,469**	**1,706**	**1,744**	**2,404**	**3,074**
Cumulative incidence	2.19%	2.25%	2.55%	2.14%	1.96%	2.34%
(CI)	(1.98–2.40)	(2.05–2.44)	(2.18–2.91)	(1.82–2.45)	(1.71–2.22)	(2.08–2.60)

*TBI* = Traumatic brain injury

*N.of* = Number of patients

*N.risk* = Number at risk

*CI* = Confidence interval

Cox regression revealed higher long-term psychotic syndrome risk in pTBI patients vs. references. Until 10 years, HR was 1.34 (CI: 1.16–1.55); during 10–18 years, HR was 1.13 (CI: 0.95–1.36). Post-18 years, risk was (HR: 1.37, CI: 0.68–2.78).

Comparing cumulative incidence differences in psychotic syndrome-free survival between female pTBI and reference groups revealed heightened risk in the pTBI group. After 1 year, cumulative incidence was 0.06% (pTBI) vs. 0.03% (reference) for females and 0.03% (pTBI) vs. 0.02% (reference) for males. After 20 years, female cumulative incidence increased to 2.55% (pTBI) vs. 2.14% (reference) (Fig. 1, Table 2).

After the initial 3 years, HR for pTBI females was 2.00 (CI: 1.24–3.23), decreasing to 1.08 (CI: 0.90–1.30) from 3 to 20 years. In males with pTBI, HR was 1.19 (CI: 0.71–1.99) post-initial 3 years, further reducing to 0.85 (CI: 0.73–0.99) during 3–20 years.

## Discussion

This nationwide study identified a link between pTBI and elevated post-traumatic psychotic syndrome risk, especially within the initial 10 years of follow-up. Notably, female pTBI patients showed a heightened risk.

The existing literature on psychotic syndromes associated with pTBI remains limited. A comprehensive meta-analysis including 8 studies and over 150,000 pTBI patients yielded findings consistent with our study, indicating a association between pTBI and psychotic syndromes, with an odds ratio (OR) of 1.80 (CI: 1.11–2.95). Notably, five of these studies focused solely on schizophrenia as an outcome. However, upon excluding two lower quality studies, the OR decreased to 1.43 (CI: 1.04–1.98), aligning more closely with our findings [3]. Similar to our study, this meta-analysis did not stratify the severity of pTBI. Additionally, subgroup analyses based on sex were not conducted in the meta-analysis. Another limitation noted in the meta-analysis was that the vast majority of pTBI patients were represented by a single study, which reported an OR of 1.21 (CI: 1.12–1.31), while the other seven studies included fewer than 1000 patients each. Another Nordic case–control study on TBI-related schizophrenia and bipolar disorder, based on over 20,000 cases, found an increased risk of schizophrenia (IRR = 1.3) and bipolar disorder (IRR = 1.3) among individuals who sustained a TBI before the age of 15, even after accounting for familial confounding [4]. These findings closely align with our results. However, it is important to note that our study included the full spectrum of psychotic syndromes, not just schizophrenia and bipolar disorder.

Our study has several strengths, particularly the high quality of the integrated registers [9, 10]. The consistent use of ICD-10 classification in Finland since 1996, coupled with standardized coding measures, enhances data reliability. Previous successful research on neurological conditions employing the Finnish Care Register for Health Care and data from the Finnish Social Insurance Institution adds credibility to our chosen data sources [11, 12]. Access to free healthcare visits for children in Finland, supported by a nationwide fee-based social insurance system, ensures equitable healthcare provision and minimizes potential socioeconomic biases [13]. Moreover, the robustness of our study is further reinforced by the substantial population size of over 70,000 patients with pTBI. Another strength of the study is the inclusion of an orthopedic fracture referencel group, which helps to mitigate confounding bias.

Our study has some limitations. Firstly, the Finnish Care Register for Health Care does not include data on patients’familial medical history, pre-traumatic psychiatric conditions, or substance misuse. This absence of important confounding factors may result in some residual confounding in our findings. Additionally, potential diagnostic inaccuracies in pTBI coding by healthcare practitioners may exist in registry data. Furthermore, by including only intracranial injuries in the TBI group, patients with isolated skull fracture diagnoses were excluded, which may have reduced the overall number of pTBI cases in our cohort. Another limitation is the use of only the S06* codes to define TBI; as a result, both the pTBI and reference groups may include patients with other TBI-related diagnoses, such as skull fractures, that were not specifically identified. Third, the study data do not encompass information from primary care, possibly leading to an underestimation of less severe injuries usually handled by primary care providers. Fourth, the registers utilized lack GCS ratings and other severity indicators, restricting the evaluation of TBI severity. Fifth, due to our limited dataset, only age and sex could be used as covariates in the analysis.

The nationwide study revealed a link between pTBI and an increased risk of post-traumatic psychotic syndrome, notably for 10-year post-trauma period, potentially attributable to post-traumatic neuroinflammation [1, 2]. This highlights the need for preventive measures for TBIs and the potential impact of extended posttraumatic monitoring or intervention in reducing the occurrence of psychotic syndrome. With increasing pTBI prevalence, extensive research on TBI-associated psychotic syndrome, involving larger cohorts and extended surveillance, is essential.

## Supplementary Information

Below is the link to the electronic supplementary material.

ESM 1 (DOCX 478 KB)

ESM 2 (DOCX 31.1 KB)

## Data Availability

No datasets were generated or analysed during the current study.
