# Supplementary material for: Pediatric traumatic brain injury and later psychotic syndromes in Finland
Source: Eur J Pediatr. 2025 May 31;184(6):380. doi: 10.1007/s00431-025-06224-3 (PMC12126354; doi:10.1007/s00431-025-06224-3)
Supplement: Supplementary file 1 — (DOCX 478 KB) [file 431_2025_6224_MOESM1_ESM.docx]

**Appendix 1. Tables containing ICD-10 Diagnose codes and operation codes used in the study, flowchart of the study population and DAG.**

Pediatric Traumatic Brain Injury and Psychotic Syndromes in Finland

Juho Laaksonen^a^_,_ Ville Ponkilainen^b^, Julius Möttönen^a^, Ville M. Mattila^a,b,c^ , Ilari Kuitunen^d,e^

^a^ Department of Clinical Medicine, University of Tampere, Tampere, Finland

^b^ Department of Orthopedics and Traumatology, Tampere University Hospital, Tampere, Finland

^c^ Coxa Hospital for Joint Replacement, Tampere, Finland

^d^ Institute of Clinical Medicine and Department of Pediatrics, University of Eastern Finland, Kuopio, Finland

^e^ Department of Pediatrics, Kuopio University Hospital, Kuopio, Finland

**Corresponding author:**

Juho Laaksonen; Department of Clinical Medicine, University of Tampere, Tampere, Finland; Arvo Ylpön katu 34, Tampere; 03 311 611; juho.laaksonen@tuni.fi

Appendix1. Diagnose codes (ICD-10 diagnostic code system).

| Diagnose code | Definition |
| --- | --- |
| S06.0 | Concussion |
| S06.1 | Traumatic cerebral edema |
| S06.2 | Diffuse traumatic brain injury |
| S06.3 | Focal traumatic brain injury |
| S06.4 | Epidural hemorrhage |
| S06.5 | Traumatic subdural hemorrhage |
| S06.6 | Traumatic subarachnoid hemorrhage |
| S06.7 | Intracranial injury and prolonged concussion |
| S06.8 | Other intracranial injury |
| S06.9 | Unspecified intracranial injury |

Appendix2. The Finnish version of the NOMESCO Classification of Surgical Procedures (NCSP)

| Operation code | Definition |
| --- | --- |
| AAD00 | Evacuation of epidural hematoma |
| AAD05 | Evacuation of acute subdural hematoma |
| AAD15 | Evacuation of traumatic intracerebral hematoma |
| AAF00 | Ventriculostomy |
| AAK80 | Partial excision of skull cap for relief of acute cerebral edema |


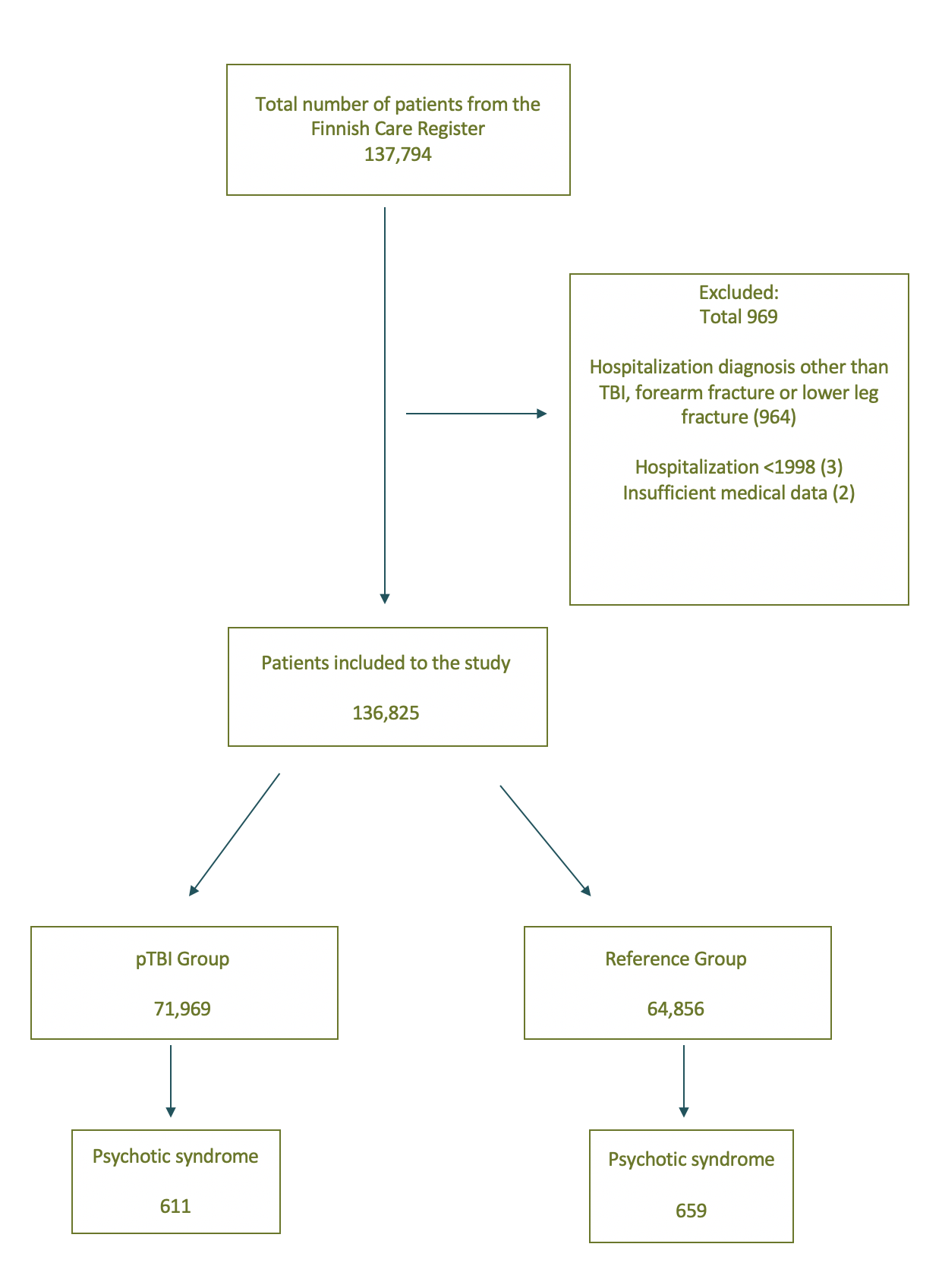


**Appendix 3. Flowchart of the inclusion and exclusion of the study population**


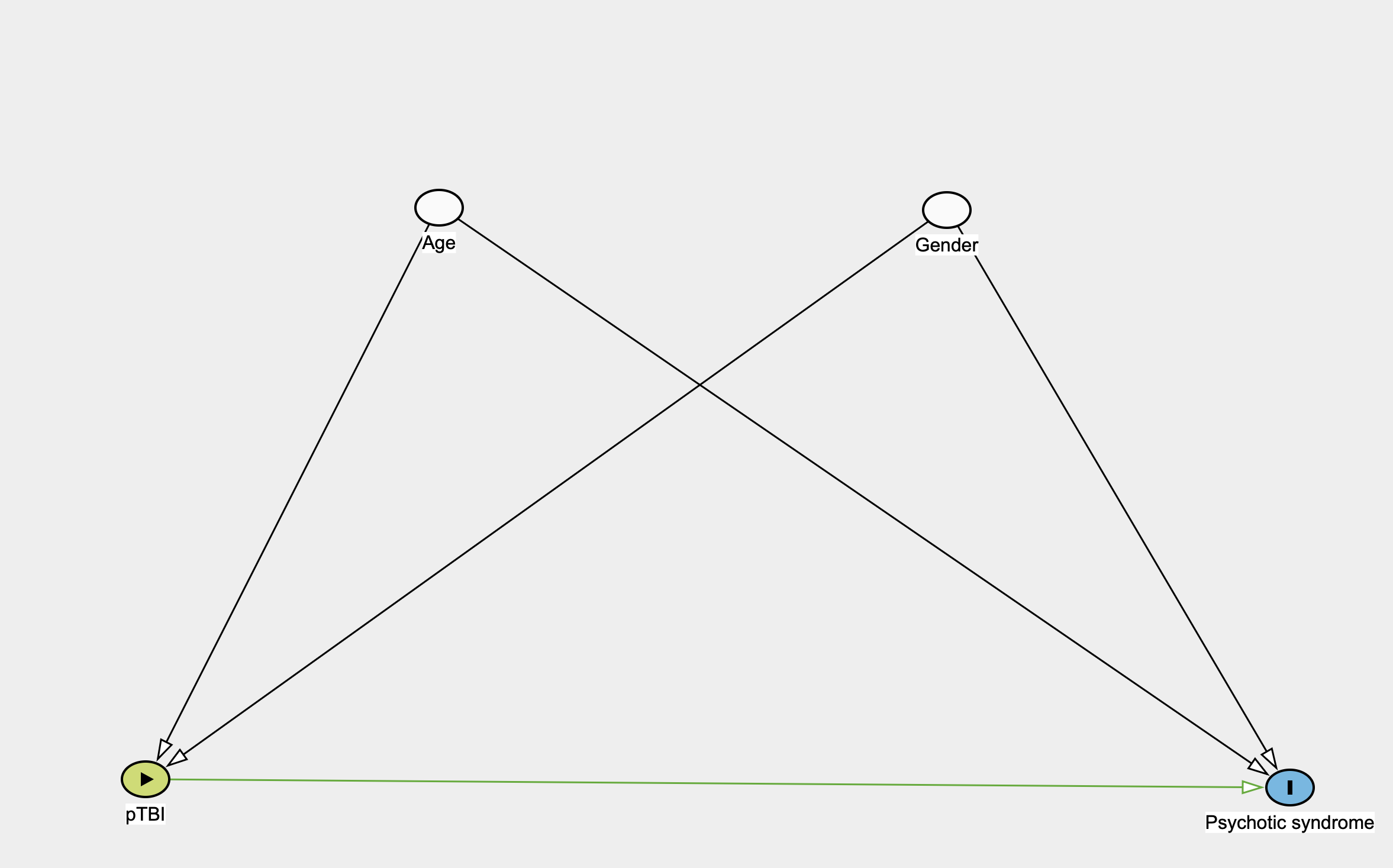


Appendix 4. Directed Acyclic Graph (DAG) illustrating the causal relationships underpinning the multivariable model examining the impact of pTBI on the occurrence of psychotic syndrome
