# Supplementary material for: Pediatric traumatic brain injury and later psychotic syndromes in Finland
Source: Eur J Pediatr. 2025 May 31;184(6):380. doi: 10.1007/s00431-025-06224-3 (PMC12126354; doi:10.1007/s00431-025-06224-3)
Supplement: Supplementary file 2 — (DOCX 31.1 KB) [file 431_2025_6224_MOESM2_ESM.docx]

STROBE Statement—Checklist of items that should be included in reports of ***cohort studies***

|  | Item No | Recommendation | Page No |
| --- | --- | --- | --- |
| **Title and abstract** | 1 | (*a*) Indicate the study’s design with a commonly used term in the title or the abstract  Register based cohort study is mentioned in title and abstract section | 3 |
|  |  | (*b*) Provide in the abstract an informative and balanced summary of what was done and what was found  Provided in abstract | 3 |
| Introduction | | | |
| Background/rationale | 2 | Explain the scientific background and rationale for the investigation being reported  Included in the introduction | 4 |
| Objectives | 3 | State specific objectives, including any prespecified hypotheses  Included in the introduction | 4 |
| Methods | | | |
| Study design | 4 | Present key elements of study design early in the paper  Included in the materials and methods section | 4-5 |
| Setting | 5 | Describe the setting, locations, and relevant dates, including periods of recruitment, exposure, follow-up, and data collection  Included in the materials and methods section | 4-5 |
| Participants | 6 | (*a*) Give the eligibility criteria, and the sources and methods of selection of participants. Describe methods of follow-up  Included in the materials and methods section | 4-5 |
|  |  | (*b*) For matched studies, give matching criteria and number of exposed and unexposed  Included in the materials and methods section | 4-5 |
| Variables | 7 | Clearly define all outcomes, exposures, predictors, potential confounders, and effect modifiers. Give diagnostic criteria, if applicable  Included in the materials and methods section | 4-5 |
| Data sources/ measurement | 8* | For each variable of interest, give sources of data and details of methods of assessment (measurement). Describe comparability of assessment methods if there is more than one group  Included in the materials and methods section | 5 |
| Bias | 9 | Describe any efforts to address potential sources of bias  Included in the limitations section in discussion | 12 |
| Study size | 10 | Explain how the study size was arrived at  Included in the materials and methods section and in Appendix3 | 4 |
| Quantitative variables | 11 | Explain how quantitative variables were handled in the analyses. If applicable, describe which groupings were chosen and why  Included in the materials and methods section | 5 |
| Statistical methods | 12 | (*a*) Describe all statistical methods, including those used to control for confounding  Included in the materials and methods section and Appendix 4 | 5 |
|  |  | (*b*) Describe any methods used to examine subgroups and interactions  Included in the materials and methods section | 5 |
|  |  | (*c*) Explain how missing data were addressed  Included in the materials and methods section and Appendix 3 | 5 |
|  |  | (*d*) If applicable, explain how loss to follow-up was addressed  Included in the materials and methods section | 4-5 |
|  |  | (*e*) Describe any sensitivity analyses  N/A |  |
| Results | | |  |
| Participants | 13* | (a) Report numbers of individuals at each stage of study—eg numbers potentially eligible, examined for eligibility, confirmed eligible, included in the study, completing follow-up, and analysed  Included in the results section | 7 |
|  |  | (b) Give reasons for non-participation at each stage  N/A due to register based study |  |
|  |  | (c) Consider use of a flow diagram  Included in the Appendix 3 |  |
| Descriptive data | 14* | (a) Give characteristics of study participants (eg demographic, clinical, social) and information on exposures and potential confounders  Included in the results section | 7 |
|  |  | (b) Indicate number of participants with missing data for each variable of interest  Included in the Appendix 3 |  |
|  |  | (c) Summarise follow-up time (eg, average and total amount)  Included in the methods and results section | 5,7 |
| Outcome data | 15* | Report numbers of outcome events or summary measures over time  Included in the results section | 7-10 |

| Main results | 16 | (*a*) Give unadjusted estimates and, if applicable, confounder-adjusted estimates and their precision (eg, 95% confidence interval). Make clear which confounders were adjusted for and why they were included  Included in the results section and table 1B and Figure 1 | 7-8 |
| --- | --- | --- | --- |
|  |  | (*b*) Report category boundaries when continuous variables were categorized  Included in the results section and table 1B and Figure 1 | 7-8 |
|  |  | (*c*) If relevant, consider translating estimates of relative risk into absolute risk for a meaningful time period  N/A |  |
| Other analyses | 17 | Report other analyses done—eg analyses of subgroups and interactions, and sensitivity analyses  Included in the results section and Table 1B and Figure 1 | 9-10 |
| Discussion | | | |
| Key results | 18 | Summarise key results with reference to study objectives  Included in the beginning of the discussion section | 11 |
| Limitations | 19 | Discuss limitations of the study, taking into account sources of potential bias or imprecision. Discuss both direction and magnitude of any potential bias  Included in the limitations section of discussion | 12 |
| Interpretation | 20 | Give a cautious overall interpretation of results considering objectives, limitations, multiplicity of analyses, results from similar studies, and other relevant evidence  Included in the end of discussion | 12 |
| Generalisability | 21 | Discuss the generalisability (external validity) of the study results  Included in the end of discussion | 12 |
| Other information | | | |
| Funding | 22 | Give the source of funding and the role of the funders for the present study and, if applicable, for the original study on which the present article is based  Funding included in the acknowledgements | 12 |

*Give information separately for exposed and unexposed groups.

**Note:** An Explanation and Elaboration article discusses each checklist item and gives methodological background and published examples of transparent reporting. The STROBE checklist is best used in conjunction with this article (freely available on the Web sites of PLoS Medicine at http://www.plosmedicine.org/, Annals of Internal Medicine at http://www.annals.org/, and Epidemiology at http://www.epidem.com/). Information on the STROBE Initiative is available at http://www.strobe-statement.org.
